# Habitat Loss, Not Fragmentation, Drives Occurrence Patterns of Canada Lynx at the Southern Range Periphery

**DOI:** 10.1371/journal.pone.0113511

**Published:** 2014-11-17

**Authors:** Megan L. Hornseth, Aaron A. Walpole, Lyle R. Walton, Jeff Bowman, Justina C. Ray, Marie-Josée Fortin, Dennis L. Murray

**Affiliations:** 1 Environmental and Life Sciences, Trent University, Peterborough, Canada; 2 Wildlife Research & Monitoring Section, Ontario Ministry of Natural Resources and Forestry, Peterborough, Canada; 3 Regional Operations Division, Ontario Ministry of Natural Resources, South Porcupine, Canada; 4 Wildlife Conservation Society Canada, Toronto, Canada; 5 Department of Ecology and Evolutionary Biology, University of Toronto, Toronto, Canada; 6 Department of Biology, Trent University, Peterborough, Canada; University of Western Ontario, Canada

## Abstract

Peripheral populations often experience more extreme environmental conditions than those in the centre of a species' range. Such extreme conditions include habitat loss, defined as a reduction in the amount of suitable habitat, as well as habitat fragmentation, which involves the breaking apart of habitat independent of habitat loss. The ‘threshold hypothesis’ predicts that organisms will be more affected by habitat fragmentation when the amount of habitat on the landscape is scarce (i.e., less than 30%) than when habitat is abundant, implying that habitat fragmentation may compound habitat loss through changes in patch size and configuration. Alternatively, the ‘flexibility hypothesis’ predicts that individuals may respond to increased habitat disturbance by altering their selection patterns and thereby reducing sensitivity to habitat loss and fragmentation. While the range of Canada lynx (*Lynx canadensis*) has contracted during recent decades, the relative importance of habitat loss and habitat fragmentation on this phenomenon is poorly understood. We used a habitat suitability model for lynx to identify suitable land cover in Ontario, and contrasted occupancy patterns across landscapes differing in cover, to test the ‘threshold hypothesis’ and ‘flexibility hypothesis’. When suitable land cover was widely available, lynx avoided areas with less than 30% habitat and were unaffected by habitat fragmentation. However, on landscapes with minimal suitable land cover, lynx occurrence was not related to either habitat loss or habitat fragmentation, indicating support for the ‘flexibility hypothesis’. We conclude that lynx are broadly affected by habitat loss, and not specifically by habitat fragmentation, although occurrence patterns are flexible and dependent on landscape condition. We suggest that lynx may alter their habitat selection patterns depending on local conditions, thereby reducing their sensitivity to anthropogenically-driven habitat alteration.

## Introduction

Populations occurring at the periphery of a species' geographic range often occupy habitats that are of lower overall quality, leading to reduced survival, reproduction and population density, compared to populations in the core of the range [1]. In addition, peripheral populations tend to be more sensitive to environmental variability than those in the core, which can promote increased demographic stochasticity and lower resilience [2]–[4]. As a result, individuals in the range periphery may be more sensitive to the processes of habitat loss and fragmentation. Alternatively, animals may respond with more flexible habitat selection patterns, enabling them to move among variable environments to enhance their fitness [5]. This flexibility should increase species' persistence in landscapes experiencing anthropogenic change, such as in areas subject to high fragmentation. However, much of our perception of how wide-ranging species respond to these landscape-scale processes is speculative, especially in peripheral populations where both occurrences and their detection probability are often limited. This shortcoming is especially relevant because as landscapes continue to be altered by anthropogenic disturbance, many species are faced with declines in range size [6]. An improved understanding of the effects of habitat loss and fragmentation on species occurrence patterns will enhance our understanding of how these processes may impact species distributions.

Habitat loss and fragmentation are separate processes whereby habitat loss is an overall reduction in the amount of suitable habitat resulting in a decline of patch size and habitat fragmentation is the breaking apart of habitat, independent of habitat loss [7]. While the effects of habitat loss on species are consistently negative, the effects of habitat fragmentation are less well understood, as few studies measure fragmentation independently of habitat loss [7]. While habitat fragmentation can have both weakly positive and weakly negative effects on biodiversity and population size, the impact of these effects is often far less important than the effects of habitat loss [7]–[9]. There is some evidence that the effects of habitat fragmentation depend upon the amount of habitat that is available in a landscape. The ‘threshold hypothesis’ predicts that individuals will be more affected by habitat fragmentation when the amount of habitat on the landscape is limiting (i.e. less than 30% habitat), and small and isolated patches become more numerous, than when habitat is abundant and patches are larger and more continuous [10], [11]. Habitat fragmentation may compound the effects of habitat loss due to changes in patch size and landscape configuration, implying that fragmentation may have a greater effect at the range periphery, where habitat is often limiting [2]. This hypothesis has been supported by several studies examining population size and presence of birds and small mammals with habitat thresholds ranging from 10–30% [10], [12]–[14]. In contrast, the ‘flexibility hypothesis’ suggests that individuals may alter their habitat selection patterns, permitting them to inhabit variable environments that would otherwise be unsuitable due to habitat fragmentation [5], [15].

Canada lynx (*Lynx canadensis*) occur across the boreal forest of North America, where their primary prey is snowshoe hare (*Lepus americanus*). Since lynx are dependent upon snowshoe hares, they select forested habitat based on high hare abundance or where they are most easily depredated [16]–[18], whereas hares select young coniferous forests where both food and cover are adequate [19], [20]. In the southern periphery of the lynx range, forest composition is more heterogeneous and hare densities are naturally lower, leading to reduced abundance and restricted distribution of lynx [21], which require densities between 1 to 1.5 hares per hectare to persist [22].Because habitat for both lynx and hare has become both reduced and fragmented due to anthropogenic activities in their southern ranges, the distribution and abundance of both species is now restricted [23], [24]. This has reduced genetic diversity in southern populations of both hare [25] and lynx [26]. Additionally, the southern range of lynx in Ontario has contracted by over 175 km since 1970 [26]. Although the mechanisms ultimately limiting lynx populations at the southern range periphery remain to be fully understood, this may be due to sensitivity to habitat fragmentation [27], with habitat loss and climate change as other important factors [26]. Several other felid species are also reported to be sensitive to habitat fragmentation (e.g. Iberian lynx (*Lynx pardinus*) [28], bobcat (*Lynx rufus*) and cougar (*Puma concolor*) [29]). However, whether these species express any flexibility in selection patterns in relation to the amount of habitat on a landscape or whether these patterns hold true for habitat fragmentation, has not yet been explored.

We examined the occurrence patterns of Canada lynx across the 2 regions in the southern geographic range of the species in Ontario to assess patterns of occurrence in relation to habitat loss and fragmentation. Given that lynx are prey specialists, requiring areas within a narrow range of suitable conditions to meet prey and habitat requirements [30] as well as connectivity requirements [31], we predicted that lynx would be sensitive to habitat loss when habitat was widely available, and sensitive to both habitat loss and fragmentation when suitable habitat was less than 30%; this would support the ‘threshold hypothesis’ [10], [11]. These patterns may be expressed more strongly near the southern range periphery, due to increased levels of habitat loss and reduced habitat quality [26], leading us to speculate that any sensitivity to habitat fragmentation would be most apparent there. Alternatively, the ‘flexibility hypothesis’ suggests that lynx will have tolerance to both habitat loss and fragmentation, such that their occurrence patterns may not correlate with either process, indicating flexibility in habitat selection. We developed a habitat suitability model for lynx and tested the above predictions using patterns of track occurrence across the species' southern range periphery. We compared two regions each with three similar levels of suitable land cover as determined by the habitat suitability model, to examine if occurrence patterns differ across landscapes with varying amounts of suitable land cover. Observations of lynx tracks in areas with limited suitable land cover and increased fragmentation would imply that lynx are not sensitive to habitat fragmentation, or that the importance of suitable habitat on occurrence patterns at the range periphery are less critical than previously understood.

## Methods

### Ethics Statement

The Trent University Research Ethics Board approved the study (reference #21083). In the introduction of the study, participants were explicitly told that informed consent was implied if they submitted their survey data. The field component consisted of non-invasive track surveys conducted on public land, so no access permits or animal care protocols were required. Canada lynx are considered not at risk under provincial and federal guidelines.

### Study Area

**Figure 1:**
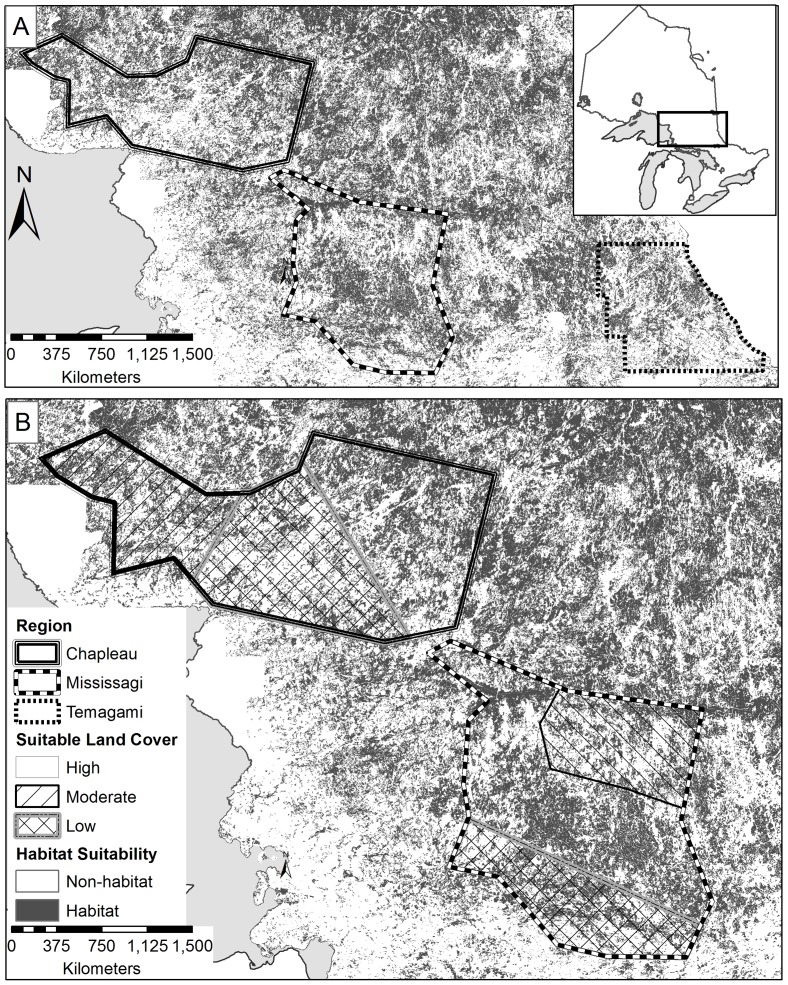
Habitat suitability map for Canada lynx in (A) central Ontario with Regions outlined and (B) suitable land cover levels within each region, as determined by the literature-based habitat suitability model.

The study area encompassed 200 000 km^2^ in central Ontario (Figure 1A), across the southern boreal forest and the Great Lakes St. Lawrence forest, a transition zone from boreal to deciduous forest, encompassing the southern range limit of lynx occurrence in the region [32]. The area is largely comprised of boreal forest, with spruce (*Picea glauca, P. mariana*), balsam fir (*Abies balsamea*), trembling poplar (*Populus tremuloides*) and white birch (*Betula papyrifera*) as dominant tree species. The southerly portions of the study area in the Great Lakes St. Lawrence region include pines (*Pinus resinosa, P. strobus*), eastern hemlock (*Tsuga canadensis*), yellow birch (*B. alleghaniensis*) and maples (*Acer saccharum, A. rubrum*). Habitat loss and fragmentation throughout the study area is caused primarily by forestry and associated road construction. Historically 1% of the entire region (approximately 2000 km^2^) was harvested annually [32], current levels are 0.04% or 800 km^2^ (2000–2010 average; [33]). Other sources of habitat loss include populated areas, agriculture, and natural disturbance such as forest fire and pest infestations.

### Habitat Suitability Model

In order to quantify lynx habitat suitability, we used the analytic hierarchy process, a decision-making procedure that is useful in the development of habitat suitability models for wide-ranging mammals (see [34], [35] for description of methodology). We developed the survey design based on a literature review identifying important ecological factors affecting lynx occurrence, with an emphasis on the southern range periphery. The primary habitat characteristics were land cover attributes (e.g., [17], [18]), forest age class (e.g., [18], [36]), annual snowfall (e.g., [37]) and road density (e.g., [38]). We developed two separate models of habitat suitability, one based on expert-opinion, where we received 11 solicited responses from lynx researchers across North America, and the other using a literature-based approach with four ‘naïve’ participants with no previous knowledge of lynx ecology. Both experts and naïve participants received the same survey and the naïve participants also received four research papers providing a detailed description of the basic habitat requirements of lynx from across its range [17], [18], [38], [39]. The survey consisted of five separate pair-wise comparison matrices based on each of the features of interest (land cover, forest development stage, snowfall, and road density) and an overall comparison of the relative importance among all features. The overall ranking of features was used to weight parameters within the model and estimate the relative importance of factors affecting lynx habitat suitability, whereas weights within a feature determined the ranking for its attributes.

We used the Ontario Forest Resource Inventory to characterize land cover; these data provide a detailed description of species composition and forest stand age as determined by aerial photo interpretation. The study area included 41 provincial forest management units, and each unit was updated with forest fire and harvest information up to and including 2008. Standardized forest units were combined to create six generalized land cover types (coniferous forest, deciduous forest, mixedwood forest, developed land, wetland, and open areas) and five forest development stages (presapling, sapling, immature, mature and old; [40]), which improved the accuracy of the dataset [41]. We converted the land cover map to a geospatial raster for analysis; all GIS analyses were conducted in ArcGIS 9.2 (ESRI, Redlands, CA, USA).

We evaluated the lynx habitat model in a portion of the study area near the North Bay - Temagami region of northeastern Ontario, Canada (47.01°N, 79.97°W; see Figure 1A). The Temagami region is approximately 8,000 km^2^ and was selected because it is located within the southern range periphery of lynx in Ontario and the transition zone of boreal forest with the northern Great Lakes-St. Lawrence forest. Between January and March 2009, we surveyed lynx occurrence at 48 randomly selected sites that represented a gradient in available land cover types [38]. We assessed lynx presence by snowtracking triangular transects around the centroid of the cell (dimensions 0.5 km per side, [38]). Additional lynx tracks that were encountered opportunistically while travelling within the landscape were also considered as lynx presence. We calculated habitat suitability at the centre of each transect and each opportunistic track, using both models. We used receiver operating characteristic plots and the Area Under the Curve (AUC) as an independent measure of model accuracy via the program ROC/AUC [42]. AUC provides a measure of model accuracy, where values >0.7 indicate good model fit. We selected P_fair_, the value where specificity and sensitivity are equal, as the threshold habitat suitable for lynx occurrence.

### Lynx Occurrence Sampling

Two regions were selected to document lynx occurrence (estimated by track identification) in landscapes across a gradient of habitat fragmentation. Each region fell within the larger study area which encompassed the southern boreal forest and Great Lakes-St. Lawrence Forest, and was divided into three landscapes based on the amount of suitable land cover (high, moderate, and low) as determined by the habitat suitability model (Figure 1B). The Chapleau region was 12 900 km^2^, located primarily in the boreal forest. The western portion of the region had the highest amount of suitable land cover and is the least fragmented landscape in this region. The central area of the Chapleau region is highly fragmented with the most habitat loss due to forestry, roads, and human settlements. The easternmost portion of this region has a moderate amount of suitable land cover and a moderate level of fragmentation due to forestry roads (Table 1). The Mississagi region was 12 800 km^2^ located primarily in the Great Lakes St. Lawrence forest. The northern portion of this region had moderate amounts of suitable land cover, but was fragmented due to forestry roads; the central portion had the highest amount of suitable land cover and was least fragmented, and the southernmost landscape had the least amount of suitable land cover in this region, with habitat loss due to forestry, human settlements and roads. These regions were surveyed for occurrence of lynx tracks from January to March 2010 and each identified track point was recorded as a lynx occurrence. All forest access roads, trails, hydro-electric line corridors, cutovers and riparian areas were sampled via snowmobile, totalling 9 320 km of survey lines in both landscapes. All lynx track locations were documented; Chapleau had 104 track points and Mississagi had 89 tracks points (see Figure S1 in Information S1).

**Table 1:** Summary of the amount of suitable land cover and habitat fragmentation across two regions in the southern boreal forest in Ontario, Canada.

Region	Land Cover^a^ Level	Area (km^2^)	Percentage of Suitable Land Cover	M_eff_ (km^2^)
Chapleau	High	5 085.7	41.88	87.31
	Moderate	3 162.8	34.95	22.41
	Low	4 639.7	20.64	5.68
Mississagi	High	7 873.2	42.84	258.61
	Moderate	3 016.8	31.85	23.14
	Low	2 356.4	25.5	18.55

a Land cover is the amount of suitable land cover measured at the landscape level as determined by the habitat suitability model.

Roads in these two regions were limited to 1 or 2 highways, <20 secondary roads, and forestry roads. To test whether there was bias arising from track proximity to surveyed roads, we randomly selected 100 points from roads (including highways, primary, secondary and tertiary roads, and snowmobile trails) and the surrounding landscape (not bisected by roads) and compared them at five spatial scales (10 km^2^, 25 km^2^, 50 km^2^, 75 km^2^, and 100 km^2^) to assess any differences in habitat quality in each region. We found that there was no difference in the amount of lynx habitat (as defined by the suitability model) in any landscape, regardless of spatial scale and distance to roads (M. Hornseth, unpublished data, but see [38]). Accordingly, we deemed that proximity of locations to roads was not relevant to our particular analysis.

True absences are difficult to detect using typical survey methods, especially without repeated visits. We randomly selected points (equal to the number of lynx locations) from survey logs to represent pseudo-absences in Chapleau and Mississagi. These locations were at least 1 km apart and at least 2.5 km from the nearest lynx location. To examine the effect of spatial scale, and to encompass overall selection patterns, we buffered both observed lynx tracks and pseudo-absences with radii of 2.82 km and 5.61 km to create areas of 25 km^2^ and 100 km^2^ (from published home range size estimates), to assess the role of spatial scale on occurrence patterns (see [18], [39]).

### Habitat Amount and Fragmentation

Landscape connectivity can be considered across a variety of spatial and ecological scales, and for our analysis the metrics of interest included estimates of: (i) structural connectivity, which represents the spatial configuration of suitable patches; and (ii) functional connectivity, which includes animal response to patches [43]. We created a binary landscape of habitat quality using the literature based habitat suitability model and a critical threshold of habitat suitability value of 52 (threshold tuned by balancing the error rate between false positives and false negatives [42]). We quantified the percentage of habitat within each lynx and pseudo-absence area to estimate habitat amount. To avoid confusion of working at multiple scales, we used the term *suitable land cover* to describe the output of the habitat suitability model at a landscape-level and *suitable habitat* to describe this output at a finer spatial scale (25 km^2^ and 100 km^2^ areas).

We used PatchMorph [44] and the habitat suitability model to estimate a ‘functionally’ connected landscape for lynx from: (1) a critical threshold of habitat suitability value of 52, (2) a minimum patch size of 5 ha (the minimum mappable forest stand (Ontario Ministry of Natural Resources, unpublished data)), and (3) a crossing distance of 200 m (M. Hornseth, unpublished data). Note that crossing distance is defined as the distance that lynx will travel in unsuitable habitat; the minimum for this metric is two raster pixels and parameters were set conservatively as per published observations of lynx habitat use patterns (see [17], [45]). Although we acknowledge that actual functional connectivity requirements for lynx are just beginning to be understood (see [46]), we consider our selected values as being within the range of those that are plausible, with minor deviations likely affecting our results only qualitatively. Additionally, we did a sensitivity analysis with crossing distances of 200 to 1000 m in 400 m increments to determine the effect of this parameter on our estimates of connectivity.

Effective mesh size can be defined as the average area potentially accessed by an animal on a given landscape without having to cross defined borders or low quality habitat, so larger values indicate that the landscape is more connected and smaller values indicate the landscape is more fragmented [44], [47]. We used effective mesh size (M_eff_) as our measure of habitat fragmentation in ArcMap 10.1 [48]. M_eff_ is calculated by:
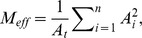
where *A* is the area of a single patch and *A_t_* can be either the total area of the polygon or the total amount of suitable habitat (i.e., the sum of all patch areas). In order to remove correlation between habitat amount and effective mesh size, we used the total amount of suitable habitat as the denominator (L. Fahrig, pers. comm.). Since correlations were still high (0.63–0.86), we regressed M*_eff_* against habitat amount and used the residuals as our estimate of habitat fragmentation (M*_eff.r_*).

### Data Analysis

We aimed to determine whether lynx are limited by habitat amount, fragmentation, or both processes, by contrasting patterns on landscapes with different amounts of suitable land cover. We hypothesized that lynx habitat requirements would restrict their occurrence to highly-connected areas in each landscape. We used one-sided unpaired *t*-tests to examine whether habitat amount and fragmentation were greater in presence areas than pseudo-absence areas at each spatial scale among landscapes with high, moderate, and low amounts of habitat amount in each region. We examined any correlations between these two within each region and landscape.

We tested 3 *a priori* hypotheses to explain lynx occurrence; i) lynx occurrence is limited only by fragmentation, ii) lynx occurrence is limited only by habitat loss, and iii) lynx occurrence is limited by both habitat amount and fragmentation. We used logistic regression and standard model selection procedures to determine which hypothesis best explained lynx occurrence in landscapes across the levels of suitable land cover. We used Akaike's information criterion to evaluate the candidate models for each lynx and pseudo-absence area and landscape within each region. We considered ΔAIC >2 to indicate a significant difference in model likelihood [49]. AIC does not assess model performance, and only models that performed well were considered plausible for the AIC model selection, so we used the Logistic Regression χ^2^ model likelihood ratio test to determine model fit.

## Results

### Habitat Suitability Model

Both the expert-opinion and literature-based models suggested that coniferous forest land cover, and forest in a sapling developmental stage, provided the most suitable habitat for lynx. However, models differed with respect to the relative importance of overall features, with the literature-based model suggesting that land cover was only slightly (1.04 times) more important than development stage whereas expert opinion suggesting that development stage was substantially (1.20 times) more important than land cover type. We omitted annual snowfall and road density from the final habitat suitability models due to low overall importance in both models (see Table S1 in Information S1).

We detected lynx at 19% (*n* = 48) of the sites within the Temagami landscape; we also included 14 more lynx track occurrences that we encountered opportunistically within the study site, increasing the total number of validation locations to 62. The literature-based model had a good overall fit (AUC: 0.912, *p*<0.001) and correctly predicted 83.9% of all sites (*n* = 62) and 82.6% of lynx occurrences (*n* = 23). The expert opinion model had a comparable fit (AUC: 0.855 *p*<0.001), correctly classifying 82.3% of all sites and 78.2% of lynx occurrences. Although both models performed well, the literature-based model surpassed the expert-opinion model in every comparison (see Table S1 in Information S1) and was selected for the remaining analyses (see Table S2 in Information S1).

### Landscape Characteristics

The landscapes within both regions had similar amounts of suitable land cover (Table 1), but different levels of habitat fragmentation. The high-cover landscape in Chapleau consisted of 41.9% suitable land cover with an effective mesh size of 87.3 km^2^. In Mississagi, the high-cover landscape had approximately the same amount of suitable land cover (42.8%), but a much larger mesh size of 258.6 km^2^. The landscapes with a moderate amount of suitable land cover in the Chapleau and Mississagi regions had similar amounts of suitable land cover (35.0% and 31.9%, respectively) and mesh sizes (22.4 km^2^ and 23.1 km^2^, respectively). The low-cover landscapes had similar amounts of suitable land cover (20.6% in Chapleau, 25.5% in Mississagi), however, the landscape in the Chapleau region was substantially more fragmented (M_eff_ 5.7 km^2^) in comparison to the matched landscape in the Mississagi region (M_eff_ 18.6 km^2^). This indicated that although the two landscapes had similar amounts of suitable land cover, generally the Chapleau landscape was more fragmented.

### Lynx Occurrence

**Figure 2:**
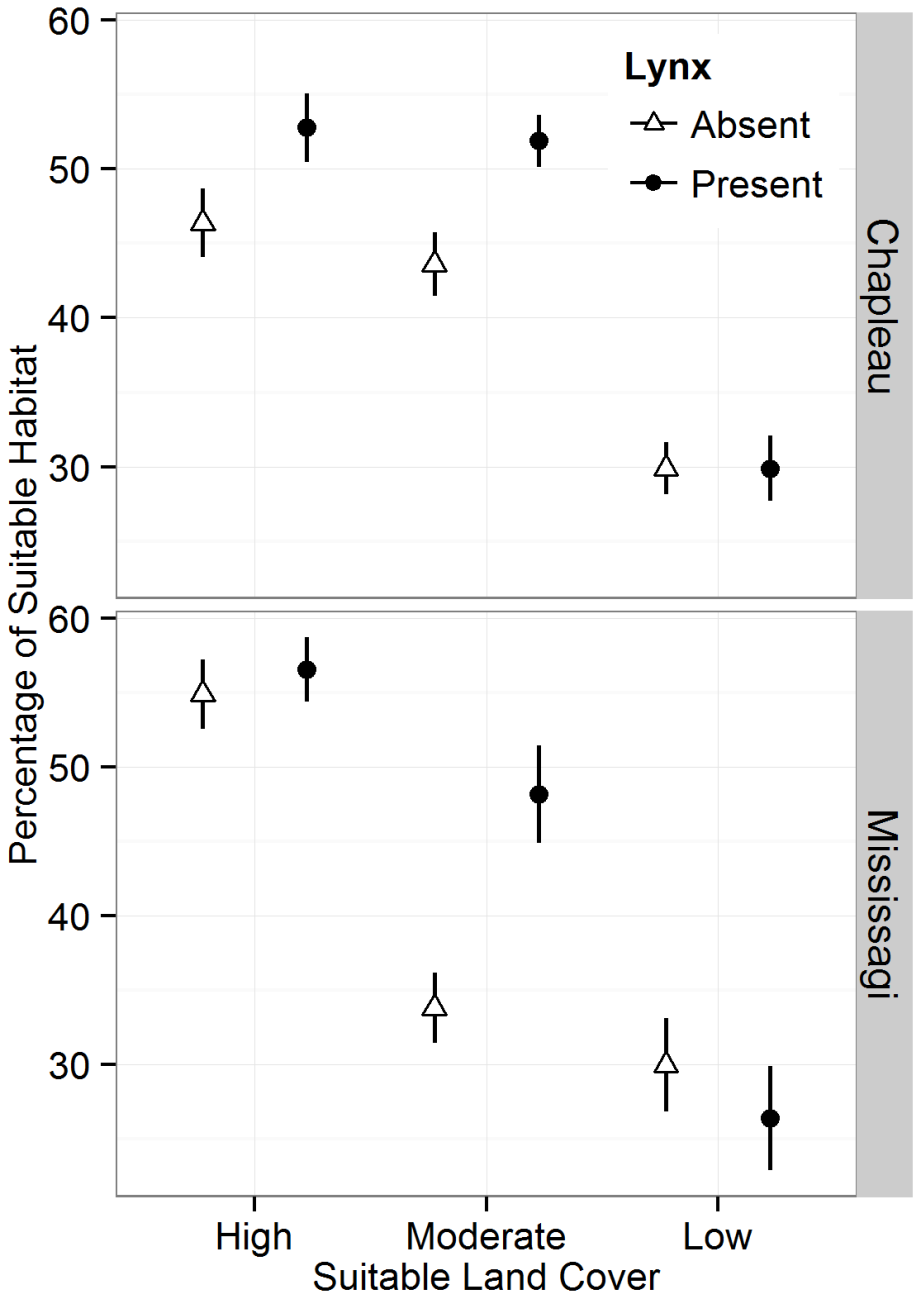
Mean percentage of suitable habitat (with standard errors) for lynx presences compared to pseudo-absences at the 25 km^2^ scale in the regions of Chapleau and Mississagi with three levels of suitable land cover.

**Figure 3:**
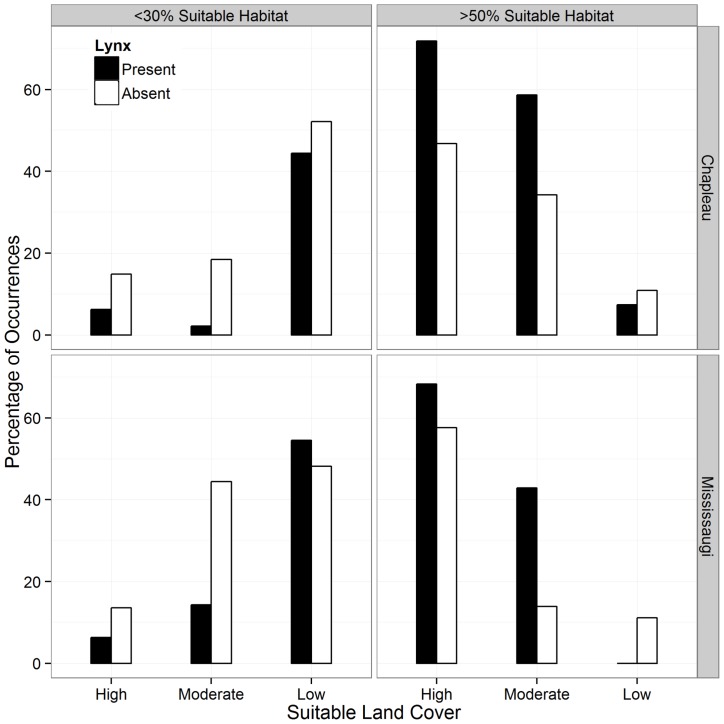
Distribution of lynx occurrences and pseudo-absences in relation to the amount of suitable habitat at the 25 km^2^ scale in the regions of Chapleau and Mississagi with three levels of suitable land cover.

Where possible, lynx selected areas with higher amounts of high quality habitat (structural connectivity) at the 25 km^2^ spatial scale (Table 2). There was a positive correlation between the amount of suitable habitat and lynx occurrence areas in both high- and moderate-levels of suitable land cover in the Chapleau region, and in the landscape with a moderate-level of suitable land cover in the Mississagi region at the 25 km^2^ area (Figure 2). In both regions, on landscapes with high- and moderate-levels of land cover, lynx consistently occurred in areas with at least 50% habitat and avoided areas with <30% habitat (Figure 3). However, in the low-cover landscapes, approximately half of lynx occurrences had less than 30% habitat at a spatial scale of 25 km^2^. These trends were consistent across both regions. At a spatial scale of 100 km^2^, there were no correlations between the amount of suitable habitat and lynx occurrence at any level of suitable land cover (Table 3). Once the effect of habitat amount was removed, there was no correlation between habitat fragmentation (M_eff.r_) and lynx occurrence on any landscape, at either spatial scale (Table 2).

**Table 2:** Summary of the differences in connectivity measures of Canada lynx occurrence and pseudo-absences in Ontario, Canada; all *t*-tests were one-sided with p-values<0.05 in bold and p-values<0.1 in italics.

Region	Area (km^2^)	Land Cover^a^ Level	Variable	Present	Pseudo-absent	*t*-test	*p*-value
Mississagi	25	High	Habitat^b^	56.56	54.90	0.53	0.300
			M_eff.r_ c	0.17	−0.20	0.76	0.223
		Moderate	**Habitat**	**48.18**	**33.8**	**3.55**	**<0.001**
			M_eff.r_	−0.77	0.45	−2.30	0.987
		Low	Habitat	26.4	29.98	−0.76	0.772
			M_eff residuals_	−0.16	0.06	−0.51	0.691
	100	High	Habitat	47.98	47.72	0.10	0.461
			M_eff.r_	−0.08	0.09	−0.26	0.601
		*Moderate*	*Habitat*	*35.35*	*31.11*	*1.33*	*0.095*
			M_eff.r_	−1.12	0.66	−1.92	0.970
		Low	Habitat	23.22	27.64	−0.97	0.829
			M_eff.r_	−0.24	0.10	−0.43	0.665
Chapleau	25	High	**Habitat**	**52.79**	**46.39**	**1.96**	**0.027**
			M_eff.r_	−0.27	0.18	−0.76	0.774
		Moderate	**Habitat**	**51.88**	**43.31**	**3.01**	**0.002**
			M_eff.r_	−0.36	0.44	−1.98	0.975
		Low	Habitat	29.87	29.88	−0.01	0.502
			M_eff.r_	0.04	−0.03	0.31	0.380
	100	High	Habitat	41.16	39.88	0.52	0.300
			M_eff.r_	−0.74	0.51	−1.29	0.899
		Moderate	**Habitat**	**39.78**	**34.75**	**2.33**	**0.011**
			M_eff.r_	−0.41	0.50	−1.30	0.901
		Low	Habitat	22.22	21.89	0.14	0.442
			M_eff.r_	−0.12	0.07	−0.96	0.829

a Land cover is the amount of suitable land cover measured at the landscape level as determined by the habitat suitability model.

b Habitat is the proportion of suitable habitat within each lynx- and pseudo-absence area based on the habitat suitability model.

c M_eff.r_ is the residual of habitat regressed against mesh size (km^2^; see text), a measure of functional connectivity, within each lynx- and pseudo-absence area.

**Table 3:** Model selection of 3 *a priori* hypotheses proposed to explain lynx occurrence patterns across 3 landscapes differing in the amount of suitable landscape-level land cover in 2 regions (Chapleau and Mississagi) within an area of 25 km^2^ for each lynx track and pseudo-absence.

	Coefficients						
Chapleau	M_eff.r_ a	Habitat^b^	AIC	ΔAIC	Weight	χ^2^	*p*-value
High Land Cover							
*Habitat Only*	*-*	0.031*	*107.2*	*0*	*0.57*	*3.61*	*0.057*
Habitat+M_eff.r_	−0.079	0.032†	108.3	1.1	0.28	4.36	0.113
M_eff_ Only	−0.072	-	110.2	3.0	0.15	0.61	0.434
Moderate Land Cover							
*Habitat+M_eff.r_*	−0.253*	0.086*	*108.7*	*0*	*0.71*	*13.00*	*0.002*
*Habitat Only*	*-*	0.053*	*110.8*	*2.1*	*0.26*	*8.85*	*0.003*
M_eff_ Only	−0.244†	-	115.7	6.0	0.03	3.98	0.049
Low Land Cover							
M_eff.r_ Only	0.098	-	100.3	0	0.43	0.06	0.802
Habitat Only	-	0.0001	100.4	0.1	0.41	0.00	0.996
Habitat+M_eff.r_	0.098	−0.0002	102.4	2.1	0.15	0.11	0.945
**Mississagi**							
High Land Cover							
M_eff.r_ Only	0.056	-	166.6	0	0.47	0.79	0.150
Habitat Only	-	0.006	166.9	0.5	0.36	0.28	0.596
Habitat+M_eff.r_	0.056	−0.006	168.4	2.0	0.17	0.87	0.329
Moderate Land Cover							
*Habitat+M_eff.r_*	−0.388*	0.073*	*63.6*	*0*	*0.87*	*17.50*	*<0.001*
*Habitat Only*	*-*	0.063*	*67.8*	*4.2*	*0.12*	*11.27*	*0.008*
M_eff.r_ Only	−0.390*	*-*	*73.3*	*9.7*	*0.01*	*5.68*	*0.017*
Low Land Cover							
M_eff.r_ Only	−0.203	-	49.6	0	0.47	0.36	0.356
Habitat Only	-	−0.016	49.7	0.1	0.39	0.45	0.511
Habitat+M_eff.r_	−0.234	0.004	51.6	2.0	0.14	0.86	0.651

Asterisk (*) indicates significant coefficients at p<0.05,

† indicates significance at p<0.1.

a M_eff.r_ is the residual of habitat regressed against mesh size (km^2^; see text), a measure of functional connectivity, within each lynx- and pseudo-absence area.

b Habitat is the proportion of suitable habitat within each lynx- and pseudo-absence area based on the habitat suitability model.

**Figure 4:**
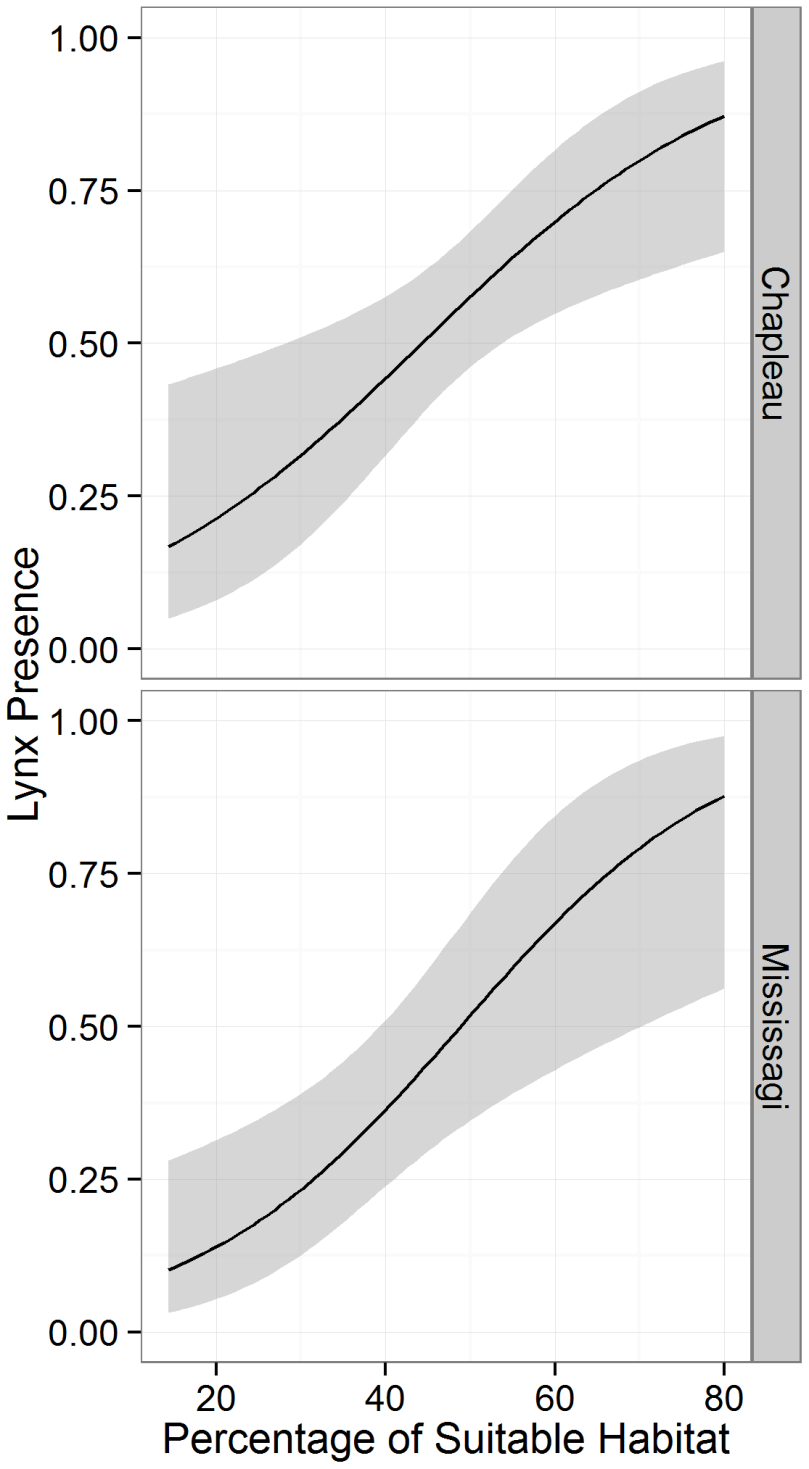
Regression plots for logistic models of Canada lynx occurrence in relation to the proportion of suitable habitat at the 25 km^2^ spatial scale in the Mississagi and Chapleau regions with moderate levels of fragmentation. The shaded area indicates the standard error.

Lynx occurrence patterns differed across landscapes, but the trends were consistent across regions. In the landscapes with moderate levels of suitable land cover, the top model included both the proportion of suitable habitat and habitat fragmentation lynx occurrence. However, only the proportion of suitable habitat had a positive association on lynx occurrence, M_eff.r_ had a negative correlation with lynx occurrence indicating that lynx selected areas with higher amounts of fragmentation (Figure 4; Table 3). In the high- and low-cover landscapes in both regions, there was no significant correlation between lynx occurrence patterns and proportion of suitable habitat or effective mesh size (Table 3).

### Sensitivity Analysis

We examined 3 crossing distances in the PatchMorph output to determine if crossing distance was either underestimated or strongly influential on lynx occurrence. We tested crossing distances of 200 m, 600 m, and 1000 m, and used standardized regression coefficients from single variable logistic regressions to determine the level of influence. Effective mesh size coefficient estimates ranged from −0.02 to 0.04, with no visible trend; none of the coefficients were significant (*p* values ranged from 0.228–0.589). Increasing the estimated crossing distance did not affect model fit.

## Discussion

Our results confirm that lynx are not sensitive to habitat fragmentation at low levels of suitable habitat, and also suggest that lynx display considerable flexibility in habitat selection patterns, supporting the ‘flexibility hypothesis’. We showed that in landscapes with moderate and high amounts of suitable land cover (30–35% and >40%, respectively), lynx occurred in areas with at least 30% available habitat and largely avoided areas below that threshold, while being unaffected by habitat fragmentation. Although this finding is consistent with the ‘threshold hypothesis’, this hypothesis also predicts that lynx would be more sensitive to habitat fragmentation on landscapes where suitable land cover was low. However, our results showed that on landscapes where suitable land cover was limited (<30%), lynx did not select areas with concentrated habitat and lynx occurrence patterns were not well correlated with either habitat amount or habitat fragmentation, instead supporting the ‘flexibility hypothesis’. Overall, we detected a threshold at which lynx occurrence patterns changed, but instead of being more sensitive to habitat fragmentation at low levels of suitable habitat, lynx displayed more flexibility in habitat selection on these landscapes. This indicates that lynx habitat choice is complex and either involves factors beyond mere resource preference, or selection of different land cover types in these areas.

### Patterns of Occurrence

As predicted by the literature-based habitat suitability model, lynx were most likely to occur in sapling-stage coniferous forest. These results are consistent with other literature on lynx habitat ecology [17], [18] and also describes snowshoe hare habitat preferences [19], [20]. Road density and annual snowfall were not important for describing lynx occurrence in Ontario. This finding contrasts with previous work (e.g., [37], [38]) but is consistent with a companion occupancy model within our study area [46], suggesting that these factors differentially affect lynx occurrence across their range and may be threshold-dependent. We surmise that low variation in snowfall patterns and low abundance of major highways as well as low road density in our study site may have accounted for the disparate results. Lynx occurrence, as determined by snow tracks across the study area, also supported this model, signifying that our model is generally robust. We recommend the use of this habitat suitability model as a tool to evaluate future forest condition on resource availability for Canada lynx in Ontario.

### Flexibility in Response to Habitat Loss

Our results suggest that when approximately 30–35% of the landscape consists of suitable land cover, there is a strong correlation between the amount of suitable habitat and lynx occurrence. While this trend was not significant at higher levels of land cover at a landscape scale, in landscapes with both high and moderate amounts of suitable land cover, lynx occurrence patterns suggest that lynx preferred areas with at least 50% suitable land cover. While lynx will occur in some areas with less than 50% available suitable land cover, lynx consistently avoided areas with less than 30% suitable habitat when suitable land cover was abundant at a landscape level. This is consistent with previous work on small mammals and birds showing that habitat occupancy dynamics are determined by species-specific tolerance thresholds [7], [11], [13].

When suitable land cover comprised only 20–25% of the landscape, our results showed that there was no correlation between lynx occurrence and habitat amount, indicating some flexibility in habitat requirements on these landscapes. In contrast, when suitable habitat was limited, lynx did not avoid areas with less than 30% land cover and were not associated with areas with more than 50% suitable habitat, despite the local availability of these areas. It is possible that when suitable habitat is scarce, lynx can survive provided that hares, or suitable alternate prey, remain available on the landscape. This speculation is supported by observations of resident snowshoe hares occupying small patches <10 ha in fragmented landscapes [50], [51] and the ability of lynx to include alternate prey items when hares are limited [31], [52]. This pattern of labile specialization has been recently documented in birds, where the most specialized species tend to generalize their habitat selection pattern following disturbance [53]. However, the results of our study contrast with previous work by Swihart et al. [5], [50], who showed that some species have greater sensitivity to habitat change at range margins. This suggests that there is a wide range of responses to habitat alteration and that further work is necessary to clarify the impact of landscape change on lynx.

### Habitat Fragmentation

Our results show that there is no correlation between lynx occurrence patterns and habitat fragmentation (M_eff.r_). M_eff.r_ (mesh size) measures the connectivity of a landscape, independent of habitat loss, so a negative coefficient indicates a positive relationship with habitat fragmentation. Our results suggest that there is a weakly negative relationship with M_eff.r_ at moderate levels of suitable land cover, which is the opposite of what we predicted. In addition, the results from our sensitivity analysis suggest that increasing crossing distance does not improve the measure of habitat fragmentation for lynx. While some studies have suggested that habitat fragmentation may only be important when habitat amount is below 30% [9]–[11], our results do not support this hypothesis. At low levels of suitable land cover there was no relationship between habitat fragmentation and lynx occurrence, which is consistent with studies showing that the effects of habitat loss are generally far greater than the effects of habitat fragmentation [7], [11]. Our results concerning habitat loss and habitat fragmentation are especially applicable to forestry-dominated landscapes, where silvicultural practices can result in marked shifts in habitat features for a variety of species, including higher densities of prey species such as snowshoe hares [54]. Therefore, we recommend that planning decisions regarding lynx consider the amount of total available habitat, which should generally improve chances of population persistence, while also benefitting overall landscape structure and function. This point is especially relevant at the southern range periphery of lynx, where habitat loss is contributing to the northward regression of the species' distribution [26].

### Conclusion

Our results highlight the importance of examining habitat fragmentation independently of habitat loss to isolate and understand the impacts of each process [7], [8]. While previous research suggests that closely related species, such as bobcats and Iberian lynx, are sensitive to habitat fragmentation [28], [29], our results show that habitat loss, not fragmentation, drives occurrence patterns for Canada lynx. The effects of habitat loss and fragmentation may be species-specific, so we recommend that this hypothesis be further evaluated in both specialist and generalist species to improve our understanding of the impacts of these wide-spread processes. This is especially necessary for carnivores, which are considered to be sensitive to both habitat loss and fragmentation [55]. Ultimately, as rates of habitat loss and fragmentation continue to increase on a global scale, this and additional research can improve conservation efforts by ensuring that recovery strategies focus on the appropriate management action.

## Supporting Information

Information S1 **Comparison of expert option and literature-based models.** **Table S1.** Performance metrics for the expert-opinion and literature based habitat suitability models for Canada lynx occurrence in Ontario, Canada. Receiver operating characteristic was based on 62 presence/absence locations near Temagami, Ontario. Bold text indicates better model performance. **Table S2.** Expert-opinion and literature based model weights for all variables used in the development of the habitat suitability model for Canada lynx in Ontario, Canada. Models were based on a survey using the analytic hierarchy decision-making process to rate the importance of different variables. The expert-opinion model is based on the replies of nine lynx researchers; the literature based model is based on the responses of 4 unbiased observers after having reviewed four research papers on lynx habitat selection. **Figure S1.** Distribution of Canada lynx occurrence across within three landscapes differing in the amount of suitable land cover as determined by a literature-based habitat suitability model in the (A) Chapleau and (B) Mississagi Regions.
(DOCX)
