# Supplementary material for: Habitat Loss, Not Fragmentation, Drives Occurrence Patterns of Canada Lynx at the Southern Range Periphery
Source: PLoS One. 2014 Nov 17;9(11):e113511. doi: 10.1371/journal.pone.0113511 (PMC4234637; doi:10.1371/journal.pone.0113511)
Supplement: Information S1 — Comparison of expert option and literature-based models. Table S1. Performance metrics for the expert-opinion and literature based habitat suitability models for Canada lynx occurrence in Ontario, Canada. Receiver operating characteristic was based on 62 presence/absence locations near Temagami, Ontario. Bold text indicates better model performance. Table S2. Expert-opinion and literature based model weights for all variables used in the development of the habitat suitability model for Canada lynx in Ontario, Canada. Models were based on a survey using the analytic hierarchy decision-making process to rate the importance of different variables. The expert-opinion model is based on the replies of nine lynx researchers; the literature based model is based on the responses of 4 unbiased observers after having reviewed four research papers on lynx habitat selection. Figure S1. Distribution of Canada lynx occurrence across within three landscapes differing in the amount of suitable land cover as determined by a literature-based habitat suitability model in the (A) Chapleau and (B) Mississagi Regions. (DOCX) [file pone.0113511.s001.docx]

**Supplemental Information: Comparison of expert option and literature-based models**

**Table S1.** Performance metrics for the expert-opinion and literature based habitat suitability models for Canada lynx occurrence in Ontario, Canada. Receiver operating characteristic was based on 62 presence/absence locations near Temagami, Ontario. Bold text indicates better model performance.

| **Metric** | **Expert-opinion model** | **Literature based model** |
| --- | --- | --- |
| Critical threshold (P_fair_) | 62 | 52 |
| AUC (SE) | 0.855 (0.046) | **0.912** (0.037) |
| p-value (AUC) | 0.0008 | **<0.0001** |
| Correct classification rate (*n*=62) | 82.3% | **83.4%** |
| Sensitivity (True Positive Rate) (*n*=23) | 78.3% | **82.6%** |
| Kappa | 0.623 | **0.661** |

**Table S2.** Expert-opinion and literature based model weights for all variables used in the development of the habitat suitability model for Canada lynx in Ontario, Canada. Models were based on a survey using the analytic hierarchy decision-making process to rate the importance of different variables. The expert-opinion model is based on the replies of nine lynx researchers; the literature based model is based on the responses of 4 unbiased observers after having reviewed four research papers on lynx habitat selection.

|  | **Expert-opinion model** | | **Literature based model** | |
| --- | --- | --- | --- | --- |
| **Attribute** | **Weight (SE)** | **Percent importance from top variable** | **Weight (SE)** | **Percent importance from top variable** |
| **Land cover** | 1.30 (0.02) | 85 | 1.68 (0.01) | 100 |
| **Forest Development Stage** | 1.53 (0.02) | 100 | 1.61 (0.01) | 96 |
| **Annual snowfall^a^** | 0.54 (0.02) | 35 | 0.21 (0.008) | 13 |
| **Road density^a^** | 0.62 (0.02) | 41 | 0.49 (0.01) | 29 |

^a^ Annual snowfall and road density were removed from the final model due to their relative low importance to lynx habitat suitability.


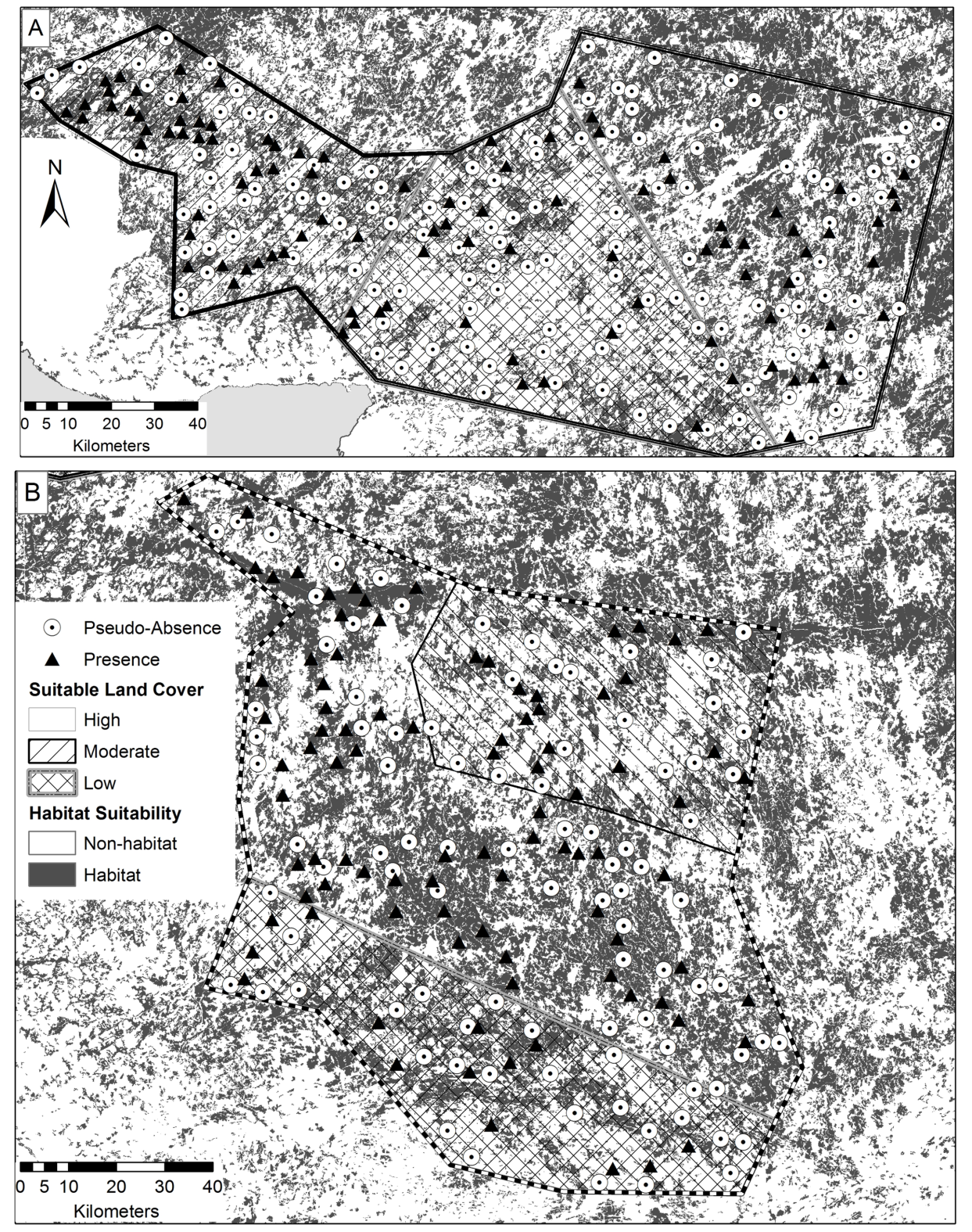


Figure S1. Distribution of Canada lynx occurrence across within three landscapes differing in the amount of suitable land cover as determined by a literature-based habitat suitability model in the (A) Chapleau and (B) Mississagi Regions.
